# Morgan County Medical Society

**Published:** 1867-11

**Authors:** Jno. W. Craig

**Affiliations:** Secretary pro tem.


### MORGAN COUNTY MEDICAL SOCIETY.

The Society met, pursuant to adjournment, at 2 o'clock P.M., at the Court House, in Jacksonville, August 8, 1867.

The President and Secretary both being absent, the meeting came to order, and was organized with Dr. Long in the chair. Dr. Craig, of Arcadia, was elected Secretary *pro tem.*

The minutes of the last meeting, as published in the *Jacksonville Journal*, were read and approved.

Drs. DeLeuw, of Jacksonville, and Edgar, of Arcadia, essayists of the day, being present, the regular order of business was taken up.

Dr. DeLeuw read an interesting paper on "Milk and Artificial Nursing." The subject being open for discussion, remarks were offered by Drs. W. S. Edgar, Prince, Lucas, Fisher, and Reed.

Dr. Edgar remarked that he thought the Doctor had made a happy choice in the selection of his theme; suggesting that practical every-day subjects were always to be preferred to mere abstract principles and theories, and as this was eminently practical, he hoped it would elicit a thorough discussion from those present.

Dr. Prince thought that the reduction of cows' milk to the constituence of that of the mothers was unnecessary—thought a millenium would scarcely ever come when there would not still be a large infant mortality; that the children of poor parents in large cities should be removed into the country, at public expense, if necessary, in order to prolong their existence by furnishing pure air, water, and better food.

Dr. Fisher remarked that his experience taught him that nearly pure cow's milk, when necessary, was the best artificial food for infants.

The generally expressed opinion of the Society was to the effect that the practice of diluting cow's milk for artificial nursing, so greatly as heretofore, was erroneous.

Dr. DeLeuw thought he had noticed scrofula as a result of too free exhibition of pure cow's milk to young infants.

Dr. Edgar observed that no rule could be strictly adopted, that each case must be separately considered, and that the rules, regulations, and statistics of the large foundling hospitals would prove highly interesting and instructive on this point.

The President, Dr. Henry Jones, of Jacksonville, with several other gentlemen of the Society, arrived.

By request, the paper of Dr. DeLeuw was re-read by the Secretary.

There being no further discussion upon the subject introduced by Dr. DeLeuw, the Society was entertained by an interesting paper read by Dr. Chas. A. Edgar, of Arcadia. The subject, cholera infantum, being one of special interest during the hot season, when such diseases are prevailing, drew forth remarks at length, from the President, and from Drs. Prince, Reed, and W. S. Edgar.

Dr. Jones endorsed and highly recommended the practice mentioned in the work of Dr. Dewees, as first put in vogue by Dr. Miller, of New York, *viz.*, one-sixth to one-fourth of a grain of the mild chloride of mercury, every hour for twelve to sixteen hours, as applicable to the acute stage, which is the stage when the treatment is most effective.

Dr. Prince, in the main, endorsed the practice referred to by Dr. Jones, and objects to the anodyne treatment in the acute stage of cholera infantum—thought that an improvement resulted from a more frequent exhibition of the mercurial in still smaller doses. He depended upon it not so much for its action on the liver as for its influence on the mucous coat of the stomach and intestinal tube. His principal objection to opiates was, that they locked up the secretions, and prevented the discharge of those principles from the blood which it is most desirable to get rid of.

In answer to a question, Dr. Prince remarked that he never observed any symptoms of cerebral disturbance following the use of the Miller treatment in acute cholera infantum.

Dr. Reed approved of the remedy, in so far as the smallness of the dose was concerned, and thought that a still greater reduction of the dose would be attended with good results; but would recommend instead, the use of camphora in small doses, frequently repeated.

Dr. Edgar, of Jacksonville, attested to the efficacy of the Miller treatment, remarking that calomel is not absorbed as calomel; that when given in small doses, it is more readily absorbed, thus producing its effect with more satisfactory results. He had obtained benefit from one-twelfth of a grain, but had failed to observe any appreciable benefit from smaller doses; that the greenish evacuations following the use of submuriate may or may not always be bile, and that the treatment would sometimes fail. He had noticed a wide difference between cholera infantum in the city and in the country; that in the country there was a greater tendency to intermittents than in the city, where the disease generally commenced with vomiting as the result of indigestion, and was attended by a low grade of typhoid fever. The doctor insisted that the use of the syringe was too much neglected; that many children had been saved by thus timely administering medicines and nourishment when articles of all kinds would be rejected from the stomach. He suggested that grated ice and ice water, in cases of excessive vomiting, were very comforting agents, and advised all young practitioners to avoid leeching and blistering, as they were not only troublesome, but often dangerous from exhaustive ulceration; finally, not to look at every case of diarrhœa among infants during the summer as cholera infantum, but to remove any cause of irritation, and rather, by timely treatment, prevent the malady.

The discussion on the subjects being over, a vote of thanks was tendered Drs. DeLeuw and Edgar for the very interesting articles furnished by them.

The President made remarks on the use of the nasal douche. "A word to the wise," etc. Also, referred to the action of tartarized antimony in certain cases of uterine inertia, claiming that it not only produces relaxation of the system generally, but positive contraction of the uterus, and related an interesting case of obstetrics, in point of theory, in which it seemed to demonstrate its power as a contracting agent.

Dr. Edgar observed that he did not see that the tartar emetic, administered as recommended, in any way hindered the operations of nature; that, whereas, it was a relaxant, he was not prepared to deny that it *might* act as a uterine stimulant. Yet he did not see but that the seeming stimulant effect was, after all, the result of nature unassisted.

The acting Secretary next read his quarterly report as Treasurer of the Society, which was, on motion, received and approved.

Dr. Baker, of Alexander Station, was unanimously elected a member of the Society.

Drs. W. S. Edgar and C. Fisher were appointed to prepare essays for the next meeting.

The Society then adjourned to meet, as usual, at 2 o'clock P.M., on the second Thursday (12th) of September.

JNO. W. CRAIG, M.D.,
*Secretary pro tem.*

